# Rheological and Mechanical Properties of an Acrylic PSA

**DOI:** 10.3390/polym15183843

**Published:** 2023-09-21

**Authors:** Beatriz D. Simões, Eduardo A. S. Marques, Ricardo J. C. Carbas, Steven Maul, P. Stihler, Philipp Weißgraeber, Lucas F. M. da Silva

**Affiliations:** 1 Institute of Science and Innovation in Mechanical and Industrial Engineering (INEGI), Rua Dr. Roberto Frias, 4200-465 Porto, Portugal; 2 Department of Mechanical Engineering, Faculty of Engineering, University of Porto, Rua Dr. Roberto Frias, 4200-465 Porto, Portugal; 3 Robert Bosch GmbH, Corporate Research and Advance Engineering, 71272 Renningen, Germanypatrick.stihler@de.bosch.com (P.S.); 4 Faculty of Mechanical Engineering and Marine Technology, University of Rostock, 18059 Rostock, Germany; philipp.weissgraeber@uni-rostock.de

**Keywords:** pressure-sensitive adhesives, material characterization, adhesive bonding, viscoelastic window, cavitation, fibrillation

## Abstract

The adhesion of pressure-sensitive adhesives (PSAs) is a complex phenomenon that can be understood through the characterization of different properties, including viscoelastic, mechanical, and fracture properties. The aim of the present paper is to determine the viscoelastic behaviour of an acrylic PSA and place it in the viscoelastic window, as well as to determine the tensile strength of the material. Additionally, different numbers of stacked adhesive layers and two crosshead speeds were applied to characterize the tensile strength of the adhesive in the different conditions. Adding a new interface between layers showed a negative influence in the tensile strength, while a higher crosshead speed implied a considerable increase in the same value. Finally, double cantilever beam (DCB) fracture tests were performed, and the J-integral approach was used to evaluate the fracture energy throughout the tests. The substrate roughness, the number of stacked layers, and the thickness of the PSA proved to decrease the performance of the PSA in fracture tests. While tensile bulk tests in viscoelastic materials are not easily found in the literature, as well as DCB tests, for fracture characterization, the obtained results allowed for the characterization of those properties in an acrylic PSA.

## 1. Introduction

Pressure-sensitive adhesives (PSAs) are characterized by forming an immediate bond with the substrate without requiring a chemical reaction, and resulting only from light pressure [1,2]. These materials have viscoelastic properties, which occur from the interaction between the van der Waals bonds that are established during bonding and the dissipative energy that is released during debonding [3]. The viscous characteristics play an important role in bond formation; yet, their elastic properties contribute significantly to energy storage during the debonding process. Thus, the ratio of these properties influences their time-dependent behaviour, as well as their strength, or resistance to removal, after joining. In their applications, these materials should provide a good mechanical strength when bonded, while maintaining their ability to easily debond [4,5,6]. There are two main viscoelastic parameters that characterize PSA behaviour: storage modulus (G′, elastic component) and loss modulus (G″, viscous component) [3,7]. PSAs should have a low storage modulus for an easy bonding process, as well as to be able to have a good adhesion to different substrates, and should present a high energy dissipation during debonding, i.e., a high value of the loss modulus [5]. In addition to the storage modulus, also the glass-transition temperature ($T_{g}$) is a critical parameter for characterizing the adaptability of PSA raw materials when being used as viscoelastic materials with pressure sensitivity [8]. These adhesives are mostly based on acrylic [9], rubber [10], silicone [11], and urethane-based polymers [12]. More specifically, acrylic-based PSAs are the most common in industrial applications, since they contain several monomers that can be modified to be adjusted to a particular function or application [13,14]. This PSA material type is composed of acrylic acid, a tackifier, if needed, and acrylic random copolymers of long-side and short-side chains, [3], which contain monomers with low and high $T_{g}$ [15]. Acrylic PSAs present several advantages, such as excellent transparency, adhesive properties, photocuring properties, weather resistance, heat resistance, and aging resistance [16]. However, this particular PSA class presents a major disadvantage, being their poor performance when bonded to low surface energy substrates [17] being the characterization of both material and system (PSA-substrate) of great importance.

The tensile characterization of the raw material PSA (bulk testing) is difficult to find in the literature, while the peel [18,19,20,21], tack [19,21,22,23,24], and shear [21,25,26] tests are the most common for characterizing the main properties of these adhesives. Huang [27] studied the mechanical performance of joints bonded with PSAs under uniaxial tensile loading, aiming to understand the effects of the configuration of the PSA-bonded assemblies, and the results show that the mechanical behaviour of these materials is very complex due to multiple transitions in the response when a carrier layer is present. Additionally, a mechanistic predictive model was developed and showed reasonable agreement with the experimental results. The peel tests are also used to relate the fracture energy and the mechanical behaviour of PSAs [28,29,30], since debonding in a peel test seems to be connected to the formation and the growth of fibrils during bond separation [28]. Another possibility for assessing the fracture energy behaviour of PSAs is the use of fracture tests, such as double cantilever beam (DCB) tests. There are some studies that use this approach, including quasi-static [31,32] and impact [33] loading conditions, although there is almost no literature on the latter. Stigh and Biel [34] established the cohesive law for a PSA tape applying the J-integral method, while studying the in-situ fracture process, for different strain rates. They obtained a cohesive law with two stress peaks, the first being related to the cavitation process and the second being associated with the stretching and failure of walls (fibrils) that were created between the cavities prior to the final fracture. They concluded that the slower rates allowed for the cavities to have more time to nucleate. Both cavitation and fibrillation are common phenomena that are observed during the debonding of PSAs from the substrate surface [32,35]. When they occur, usually the failure process can be separated in three phases: the homogeneous deformation, the nucleation and growth of cavities, followed by fibril formation and fibril elongation and breakage [35]. Finally, the maximum stress is determined by cavitation, while fibrillation should control the maximum strain at failure [36].

In the present paper, different rheological and mechanical properties are determined, considering several conditions. The study presents two main goals: to understand the influence of the thickness of the bulk specimens in stacked layers and the crosshead speed in the tensile strength of the PSA, and to investigate the influence of different conditions, both in the substrate and the adhesive layer, in DCB tests and, consequently, in the fracture energy behaviour of the adhesive. Moreover, G′ and G″ values obtained from the rheological tests allow for the positioning of the acrylic PSA in the viscoelastic window, which enables a deeper knowledge about the viscoelastic behaviour of the material.

## 2. Experimental Details

### 2.1. Materials

In the present study, an adhesive transfer tape with a high-performance acrylic adhesive was characterized. This material presents properties such as long-term strength and temperature stability. Considering that it allows for joining of different materials in several adverse environments, it can be used for industrial applications. The manufacturer’s datasheet indicates a Young’s modulus of $4.5\times 10^{-1}$ MPa and a Poisson’s ratio of 0.499 for this adhesive.

Transparent polymethyl methacrylate (PMMA) was used to manufacture the substrates used in the DCB specimens. This material presents a Young’s modulus of 3 GPa and a Poisson’s ratio of 0.35 [34]. The use of this material allowed for a better understanding of the fracture process of the PSA in the DCB tests, as it enabled the ability to visually observe the damage process throughout testing, considering its transparency properties.

### 2.2. Specimen Manufacturing

#### 2.2.1. Bulk Specimens

**Figure 1:**
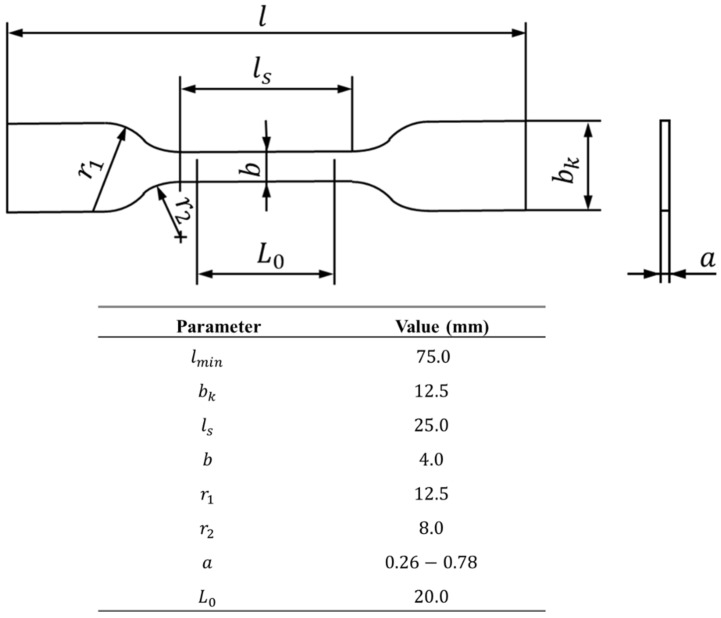
Bulk specimen’s geometry (**top**) and the respective values in mm (**bottom**).

Standard DIN 53504 [37] was followed for the manufacturing of the dog bone bulk specimens of the PSA. Figure 1 depicts the schematic representation of the specimens used to characterize the tensile behaviour of the acrylic adhesive. The material was supplied in sheets with a pre-set thickness, which implied an adaption regarding the used standard. In the present work, the effect of the number of stacked layers in the tensile strength was evaluated, and thus, the thickness of the specimens varied between one, two, or three stacked layers.

Since the acrylic PSA used was supplied in sheets, the material had to be cut in accordance with the final geometry. To accomplish this, a CAD model was created, and the specimens were then cut out using the 3D-printed part as a precise cutting guide.

After attaining the desired geometry, aluminium tabs were added to the ends of the specimens. This step was required in order to attach the specimens to the gripping system of a universal testing machine.

#### 2.2.2. DCB Specimens

**Figure 2:**
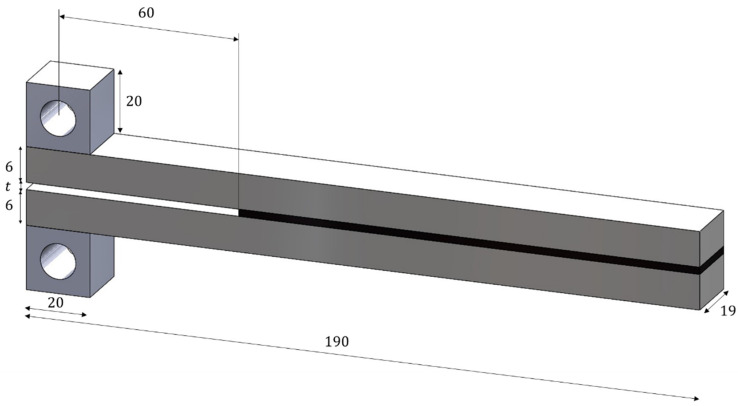
DCB specimens’ geometry in mm.

The DCB specimens were used to perform fracture tests in order to obtain the fracture energy of the acrylic PSA, for different conditions. The PMMA substrates were first cleaned with isopropyl alcohol to eliminate residues and grease that might be present in the material and can have a negative impact on the adhesion process. Afterwards, two loading blocks were bonded to the substrates, to allow for the measurement of the rotations at the loading point. Finally, the specimens were assembled with the acrylic PSA in a mould with 23 kPa applied to each joint and then resting for 72 h before being tested. Figure 2 represents the DCB specimens’ geometry, based on the work of Stigh and Biel [34], where t is the thickness of the adhesive tape.

The effect of the thickness was evaluated, and the adhesive layers varied from 0.13 mm, when the thinner adhesive series was used, to 0.26 mm per layer for all the other test configurations. The length of the DCB bonded with the PSA, 130 mm, together with a 60 mm initial crack length, is long enough to ensure that the end of the specimen is considered to be stress free, which is a mandatory condition for the approach used to measure the fracture energy.

### 2.3. Testing Procedures

#### 2.3.1. Roughness Determination

In the present work, the PMMA substrates for DCB testing were used in three conditions: untreated material and the surface abrased with P150 and P60 sandpaper. As so, the average roughness of the samples was determined to further analyse its influence on the results. 

The roughness of the neat material was determined at CEMUP (University of Porto), resorting to atomic force microscopy (AFM). The tests were performed with a Veeco Multimode Nanocsope IVa (New York, NY, USA) using the tapping mode at 316.3 kHz, with a Bruker TESP-V2 tip. The NanoScope 6.13 software was used to analyse the results.

For the abrased surfaces, a rugosimeter was used to determine the roughness profile of the substrates. The tests followed ISO 1997 standard and were performed with a Mitutoyo SJ-140 (Kanagawa, Japan) with a Gaussian filter, cut-off wavelength $\lambda_{c}=0.8\;\mathrm{mm}$, and a bandwidth wavelength $\lambda_{s}=0.5\;mm$. Five readings were executed for each sample, with a reading speed of 0.5 mm/s with the contact tip covering 4.80 mm.

#### 2.3.2. Surface Energy

The surface energy of the PMMA substrates with and without the application of plasma to the surface was determined by first measuring its wettability. The contact angle determination was carried out at room temperature with an OCA 15 goniometer (DataPhysics, Neurtek Instruments, Eibar, Spain) and three different liquids: water (polar liquid), ethylene glycol 55% (polar liquid), and n-hexadecane (nonpolar liquid). Three measurements were performed in a 30 s time interval, and the average of the obtained values was considered to further estimate the value of the surface energy. Finally, the surface energy of the material was determined, resorting to the Owens–Wendt–Rabel–Kaelble (OWRK) method [38].

#### 2.3.3. DMA Measurements

To determine the $T_{g}$, a dynamic mechanical analysis (DMA) was performed with a Netzsch DMA 242 E Artemis (Erich Netzsch GmbH & Co. Holding KG, Selb, Germany) testing machine in tension deformation mode. Rectangular samples measuring 20 × 5 × 0.26 mm were prepared, and the tests were performed at 1 Hz and a constant amplitude of 40 μm, through a temperature range from −100 °C to 15 °C. The temperature sweep was conducted at 2 K/min.

#### 2.3.4. Rheological Measurements

To study the viscoelastic behaviour of the used acrylic PSA, the storage modulus, G′, and the loss modulus, G″, were registered for different frequencies, more specifically, to determine its position in the so-called Chang’s window [39] or viscoelastic window. According to the position of the material in this window, it is possible to attribute some properties and applications of the material. The rheological properties were determined using a Kinxus Pro rheometer (Malvern Instruments, Malvern, UK) at 25 °C. For the oscillatory measurements, parallel plate geometries were used. The linear viscoelastic region (LVR) of the samples was determined using 8 mm diameter sandblasted parallel plate geometries, compressing the samples (oscillatory measurement gap), and iteratively performing strain amplitude sweep and frequency sweep measurements. The strain amplitude sweeps were performed between 0.01 and 100%, with a fixed frequency of 1 Hz. The frequency sweeps were performed over a range between 0.01 and 100 Hz, at a fixed shear strain of 1%, where the condition for the LVR was ensured.

#### 2.3.5. Bulk Tensile Testing

The bulk tensile tests were performed on a twin-column tensile tester Mecmesin^®^ MultiTest-10i. Depending on the tested conditions, the machine was equipped with a 10 N or a 500 N load cell, and the stress–strain curves were recorded for each test until the failure of the specimen. All specimens’ dimensions were measured before each test, ensuring the accuracy of the tensile stress measurements. The tests were performed at two constant crosshead speeds of 15 and 225 mm/min in order to determine the tensile strength of the adhesive and its sensitivity to different strain rates. The specimens were attached to the machine resorting to clamps that held both ends of the specimen through aluminium tabs that were previously added. Since the determination of the engineering stress and strain values assumes that the cross section of the specimen remains constant throughout the test, also the true stress and strain values were obtained. This assessment allowed for evaluating the range of values where this assumption is valid, according to Equations (1) and (2).

(1) $$\sigma_{true}=\sigma \cdot \left(1+\varepsilon_{eng}\right)$$

(2) $$\varepsilon_{true}=ln\left(1+\varepsilon_{eng}\right)$$

where σ stands for the engineering stress and $\varepsilon_{eng}$ represents the engineering strain. The engineering stress was obtained dividing the load by the initial cross section, and the engineering strain was determined considering that the deformation would be the displacement of the machine divided by the original gauge length of the bulk specimen, $L_{0}$. For bulk testing, at least three specimens were tested in order to ensure the repeatability of the results.

#### 2.3.6. DCB Testing

For the testing of the DCB specimens an INSTRON^®^ 3367 (Illinois Tool Works, Hopkinton, MA, USA) universal testing machine was used, equipped with a 30 kN load cell. The specimens were assembled in the testing machine by connecting the loading blocks to the machine attachment elements with stiff steel pins. All tests were performed in a displacement-controlled mode, at a constant crosshead speed of 2 mm/min. For further calculation of the fracture energy, the rotations on the loading points were recorded resorting to Sick^®^ TMS22E-PKH080 sensors (Waldkirch, Germany), and were synchronized with the universal testing machine.

The loads and angles measured during the tests were used to apply the J-integral approach to determine the fracture energy for each test. Given a mode I loading, the value of J along the DCB specimen’s exterior boundary is given by [40]:

(3) $$J_{I}=\frac{P}{b}\cdot (\theta_{up}-\theta_{low})$$

where P is the applied load, b is the width of the specimen, and $\theta_{up}$ and $\theta_{low}$ are the relative rotations of the upper and lower substrates at the loading points, respectively. This relation is valid, considering the path independence property of the J-integral for linear and nonlinear elastic materials, and assuming that the region outside the fracture process zone (FPZ) remains elastic during fracture [41]. For DCB tests, at least three specimens were tested to ensure the repeatability of the results.

## 3. Results and Discussion

### 3.1. PMMA Roughness

**Figure 3:**
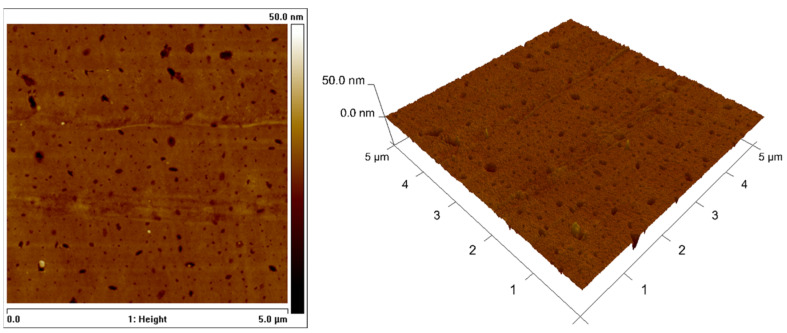
Roughness profile of the neat PMMA substrate: 2D image (**left**) and 3D image (**right**).

The AFM analysis results for the untreated substrates can be observed in Figure 3, where a low roughness surface is presented. The smooth roughness profile of the neat PMMA sample was interspersed with some nanosized grooves.

The other two samples were expected to present higher values of average roughness, given that the use of sandpaper considerably abrased the PMMA surfaces. Table 1 presents the comparison of the three conditions.

By comparing the values of $R_{a}$, the average roughness, and $R_{q}$, the root mean square roughness, for all the surfaces, it can be concluded that the abrased surfaces presented much higher values, more than 1000× for the first parameter and more than 800× for the latter. When analysing the results between the sanded surfaces, the difference is much smaller, and the surface sanded with P150 sandpaper shows lower values for all three parameters.

Since the profile was obtained using two different techniques, there are two parameters that are not presented for all the conditions; however, it is still possible to compare them. The parameter $R_{z}$ is the arithmetic average of the absolute values of the ten deepest valley depths at the surface in relation to an average line, and $R_{max}$ is the maximum absolute value of the deepest valley depth at the surface area, analysed with AFM. Considering that the standard deviation of $R_{z}$ is very low and that $R_{max}$ has a value around 200× lower, it can be stated that the maximum values for the neat surface are very low when compared to the abrased surfaces.

### 3.2. Surface Energy

**Figure 4:**
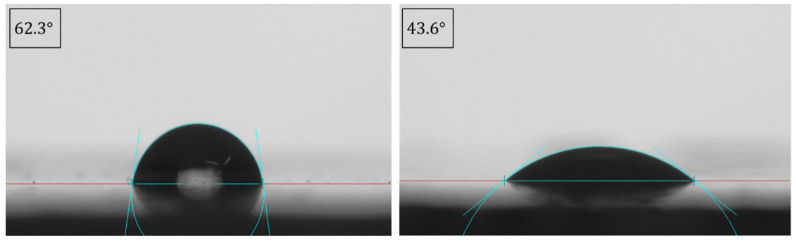
Contact angle measurement of water in the surface before (**left**) and after (**right**) the plasma treatment.

The use of plasma treatment to increase the surface energy of polymers and therefore improve the adhesion between a substrate and an adhesive layer is widely used. In the present work, a device from Arcotec GmbH (Ebhausen, Germany) was used to create a high-voltage spark discharge, which, through airflow, is transferred from the electrode to the surface, increasing the surface energy [42]. After a treatment of 15 s of plasma throughout the surface, it was expected that the contact angle between the liquids and the surface decreased, and consequently, the wettability increased. Figure 4 depicts the difference between the contact angle of a water drop in the surface before and after the treatment.

After the plasma treatment, all the three liquids presented a decrease in the contact angle values, and therefore, it can be concluded that the plasma treatment improved the wettability of the surface. Table 2 presents the values for the different liquids.

Finally, the surface energies could be calculated, and as expected, the specimens with no plasma treatment presented a lower value of 38.37 mJ/m^2^ and the ones with the plasma treatment achieved a value of 54.12 mJ/m^2^.

### 3.3. Rheological Properties

**Figure 5:**
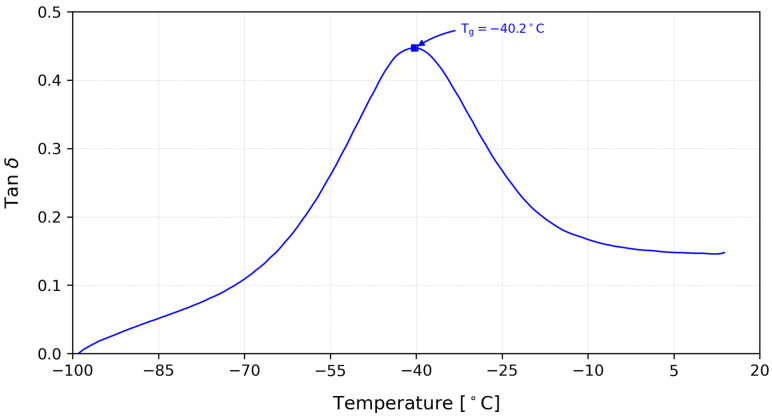
Curve obtained from the DMA analysis and the respective $T_{g}$ value.

The acrylic PSAs are composed of different types of acrylic monomers: one with high $T_{g}$, and another with a low $T_{g}$ and a functional monomer. Typically, acrylic PSAs contain 50–90% of the low $T_{g}$ monomer, 10–40% of the high $T_{g}$ monomer, and 2–20% of the functional monomer [8]. The final value of the material $T_{g}$ is determined by the balance between the different monomers. Figure 5 shows the curve obtained from the DMA analysis and the value of the $T_{g}$ for the PSA.

The value of the $T_{g}$ was determined by identifying the peak value of the tan *δ* curve, which is a loss–elasticity ratio, as well as a measure of the damping. The acrylic PSA presented a $T_{g}=-40.23\;{}^{\circ}C$, which is a common value for this kind of material [8].

**Figure 6:**
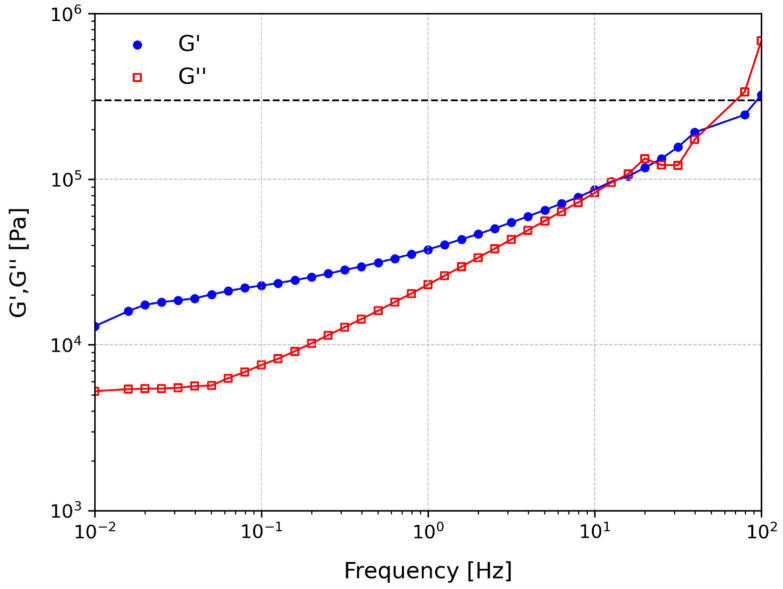
G′ and G′′ curves of the acrylic PSA as a function of the frequency.

Figure 6 shows the curves of the storage modulus, G′, and loss modulus, G″, as a function of the frequency. For the higher frequencies some fluctuation was detected due to the viscous nature of the material, which implies a delay in the response when loaded, which becomes more evident for higher frequencies in shear.

For most of the curve, G′ presents higher values than G″, indicating a predominantly elastic behaviour of the PSA for these frequencies, and high values of G″ in the zone corresponding to the debonding of the adhesive layer (>10^2^ Hz). A PSA with a lower G′ at high frequencies will present high shear performance and will be easy to peel, and a high G′ at low frequencies will have good resistance to creep or cold flow. Additionally, a high value of G″ in the debonding region is related to an increase in energy dissipation, promoting adhesive strength. The dashed horizontal line in Figure 6 represents the Dahlquist criterion, which establishes that the limit for G′ in bonding is 3 × 10^5^ Pa [43]. Hence, the values of G′ should be lower than this value at 1 Hz in order to ensure that the PSA has enough tackiness to conform to the substrate in a short time.

**Figure 7:**
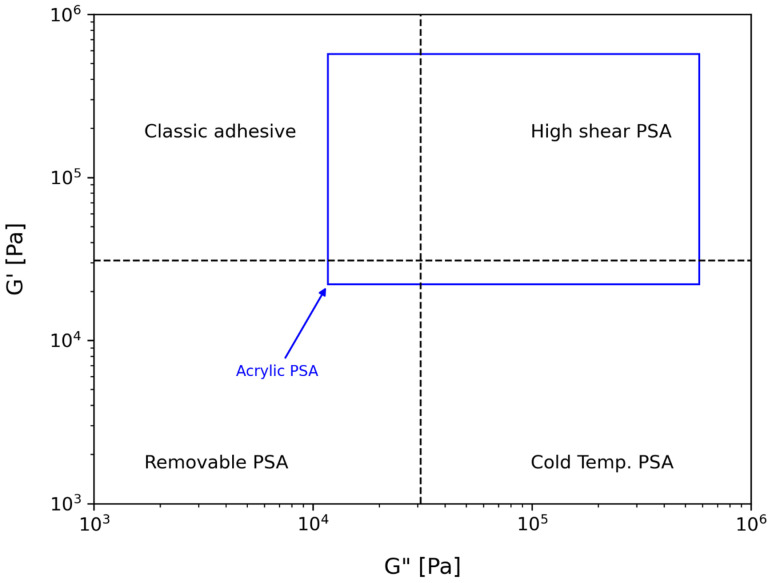
Viscoelastic window (VW) for the acrylic PSA.

The viscoelastic behaviour of the acrylic PSA was characterized by resorting to the concept of the viscoelastic window, presented in Figure 7, a concept that was developed by Chang [39] and allows for the determination of the application of a PSA. From the four coordinates (G′ and G″ at 0.01 and 100 Hz), the viscoelastic window of a material can be determined and placed within the different quadrants, in which the PSA behaviour is established.

Analysing Figure 7, it can be observed that the acrylic PSA is located mostly in the high shear quadrant. These adhesives present very high shear and moderate peel resistance. The materials located in this quadrant are used in high-performance tapes or in outdoor applications. However, the PSA also presents a region in the non-PSA quadrant, where materials have high elasticity, reduced adhesion, and easy debonding. 

### 3.4. Bulk Tensile Tests

**Figure 8:**
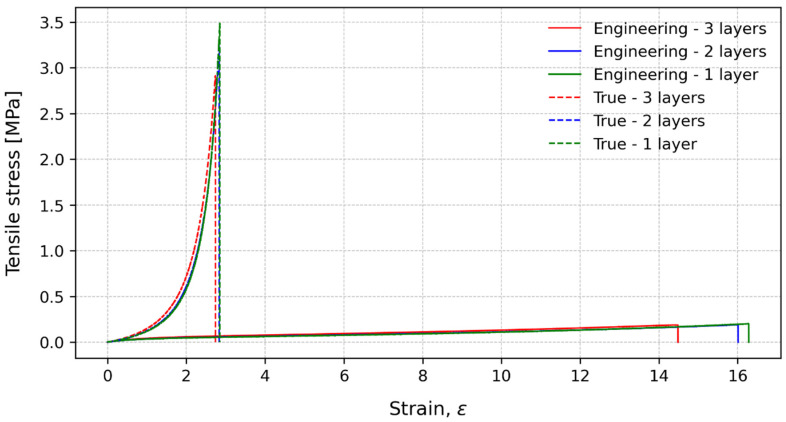
Characteristic stress-strain curves for engineering values (solid lines) and true values (dashed lines) at 15 mm/min.

The bulk tests were performed at two crosshead speeds and different numbers of stacked layers to understand the PSA sensitivity to different strain rates, as well as the thicknesses and presence of interfaces. The characteristic stress–strain curves for 15 mm/min are presented in Figure 8. For the engineering strength, the number of layers seemed to have a small influence, although a slight increase in the strength could be observed as the number of layers decreased. Regarding the strain to failure, there is a visible increase when the number of layers is decreased. For the true tensile strength, the difference in the tensile strength, when comparing the number of layers, is more pronounced. The specimens with only one layer presented the highest value, while the specimens with two and three layers presented more similar results. This could indicate that the material is more sensitive to the introduction of a new interface than the number of interfaces. The introduction of a new interface adds a new weaker point for the specimen to failure. So, regardless of the number of layers, it will always fail in one of the interfaces introduced by the stacking process. The true strain to failure considers the real strain of the specimen, and the values for the three conditions were collapsed in about the same values.

**Figure 9:**
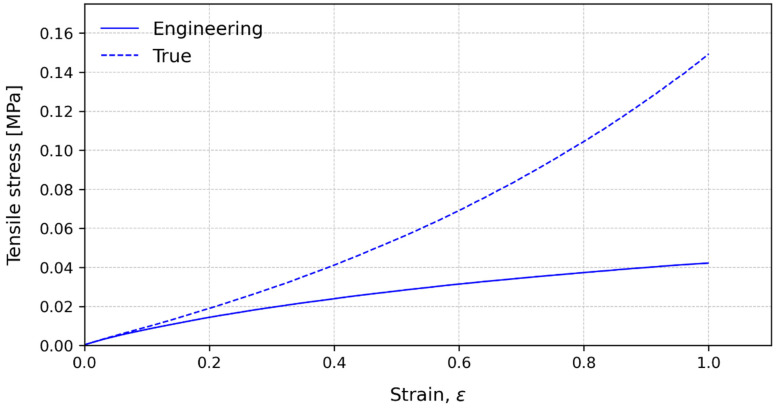
Characteristic stress–strain curves for engineering values (solid lines) and true values (dashed lines) at 15 mm/min until 100% strain for the three-layer configuration.

Figure 9 shows the characteristic stress–strain curves for 15 mm/min until 100% strain is reached, for the three-layer configuration. For very small strains, the curves are coincident until 2% and show similar results, with a difference less than 10% in stress and up to 10% of strain. This indicates that, for these strains, the values of engineering stress can be assumed to be real and used to characterize the material behaviour or for design purposes.

**Figure 10:**
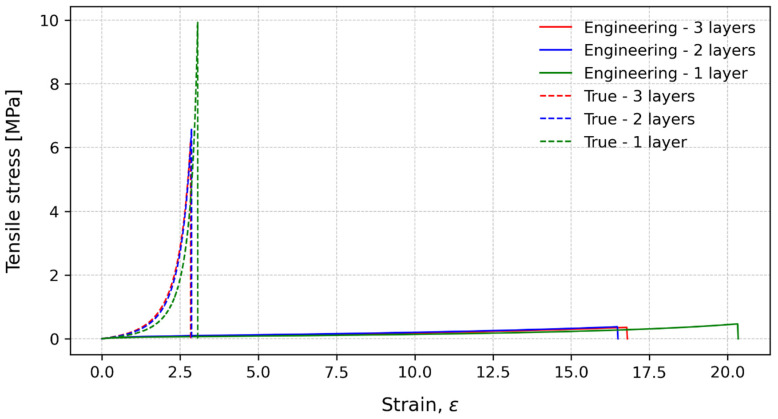
Characteristic stress–strain curves for engineering values (solid lines) and true values (dashed lines) at 225 mm/min.

The characteristic stress–strain curves for 225 mm/min are presented in Figure 10. As for the 15 mm/min condition, the number of layers seem to have a small influence on the engineering tensile strength, with a minor increase when decreasing the number of layers. When analysing the strain to failure, a decrease in the number of layers implies a significant increase in the strain to failure. Regarding the true tensile strength, the specimens with one layer show the highest value, with a big increase when compared to the two and three layers. For an increase in the crosshead speed, and therefore, an increased strain rate, adhesives tend to present a more brittle behaviour and are more prone to failure due to defects. In the present study, the crosshead speed was increased fourteen times, resulting in a true tensile strength almost three times higher. Additionally, the same trend for 15 mm/min was observed for the influence of the number of layers. It seems that introducing one new interface has a bigger influence than the number of interfaces of the specimen.

**Figure 11:**
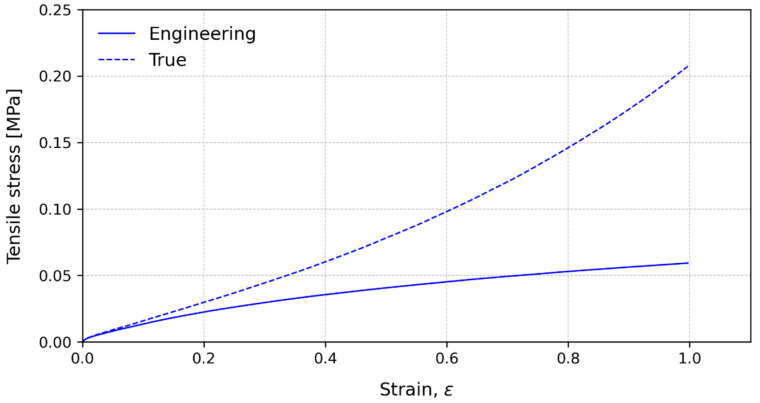
Characteristic stress–strain curves for engineering values (solid lines) and true values (dashed lines) at 225 mm/min until 100% strain for the three-layer configuration.

Figure 11 shows the characteristic stress–strain curves for 225 mm/min until 100% strain is reached for the three layers configuration. As in the slower speed, for very small strains, the curves are coincident until about 2% and show similar strain results, with a difference of less than 10% in stress and up to 10% of strain. Thus, for these strains, the values of engineering stress could be assumed to be real and used to characterize the material behaviour in this condition.

By comparing Figure 9 and Figure 11, it can be observed that, for the same values of strain, the results of the 225 mm/min tests present higher values of stress. This result is in accordance with the general behaviour of the PSA when the crosshead speed and therefore the strain rate increases. Nonetheless, for small strains, both conditions behave similarly when comparing engineering and true stress, and depending on the application, the engineering values could be used until the same value of 10% strain is reached.

### 3.5. DCB Tests

**Figure 12:**
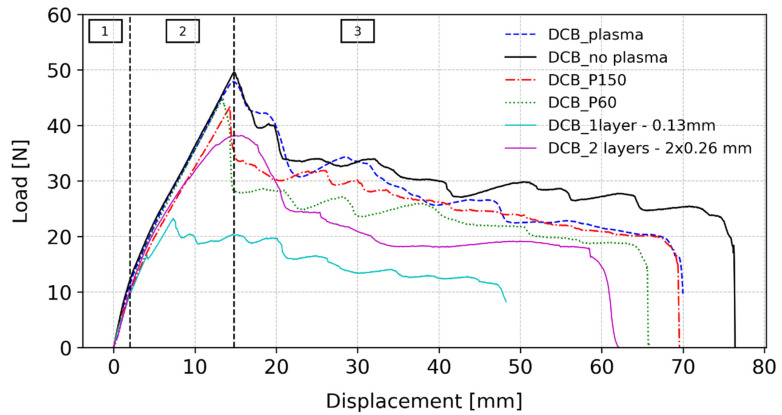
Load vs. displacement curves obtained in DCB tests for the different tested conditions.

A representative curve of the DCB test results for the six tested conditions can be observed in Figure 12. Although some differences in the failure displacement and failure load can be depicted in the load–displacement curves, for the different testing conditions, all the curves present a similar behaviour. Initially, the load increases virtually linearly, corresponding to the substrate linear elastic behaviour, and at this stage of the test, the characteristics of the substrate PMMA dominate the behaviour of the joint. This stage corresponds to phase 1, which can be observed in Figure 12, and was marked for the reference specimen for a better understanding of the described phenomena. At higher loads, the PSA’s non-elastic behaviour becomes apparent, and the curve’s behaviour changes to a different slope that corresponds to phase 2. This second phase comes to an end when damage appears and begins to propagate. Following that, the load reduces, although it shows some variations until failure, which can be observed in phase 3. The same behaviour was observed by Stigh and Biel [34], including the three phases described.

**Figure 13:**
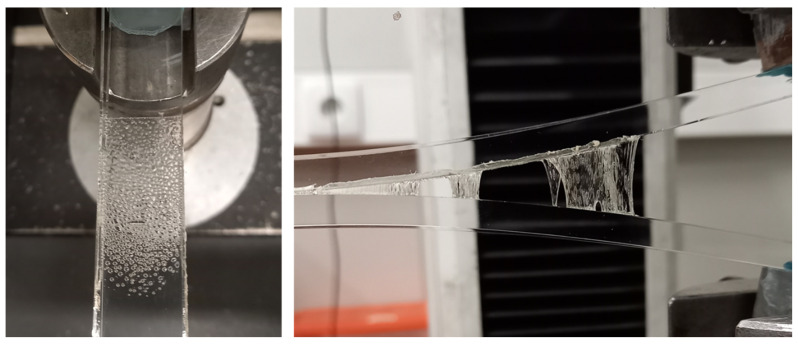
Formation of cavities (**left**) and fibrils (**right**) during a DCB test.

For all the tests, the phenomena of cavitation and fibrillation could be observed, as is represented in Figure 13. The nucleation, growth, and coalescence of the cavities in the interface PSA–substrate, are dependent on the relation between the surface tension and the Young’s modulus [32] and can be observed in Figure 13 left. This growing and stretching continues until walls form between adjacent cavities and ultimately break in a larger crack surface. After this stage, it is possible to observe the fibrillation phenomenon where there is a bridging of PSA between the substrates (Figure 13 right). The cavities start to form in the beginning of the test, and a decrease in the geometric confinement of the adhesive layer occurs, leading to a lower hydrostatic stress state. However, the formation of fibrils contributes to the increase in the deviatoric stress and allows for a hardening of the layer. The final stress state in the PSA is a balance between the two stress components and depends on the cavitation and fibrillation growing throughout the layer [44].

**Figure 14:**
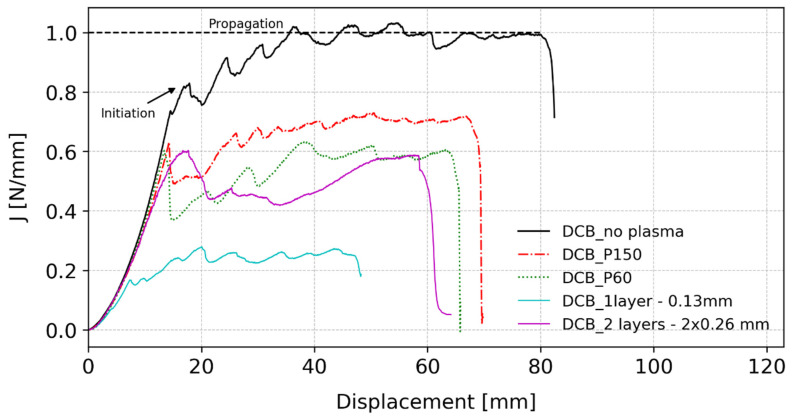
J vs. loading point displacement curves for the different tested conditions in DCB tests.

After testing the DCB specimens, the J-integral data reduction method [41] was used to assess the fracture energy for the different conditions, resorting to Equation (3), whose representative curves are shown in Figure 14. All the curves present a similar behaviour, with a parabolic part at the beginning of the tests followed by an almost linear part that lasts until the point that corresponds to the failure load in the load–displacement curves, indicated as ‘Initiation’ for the reference specimen in Figure 14. At this stage, the PSA already presents crack surfaces and there is damage in the adhesive layer. After the described initiation, the remaining part of the curve corresponds to the test stage where the cavitation and fibrillation areas are increasing and the adhesive is losing its cohesive capacity. The variations observed in all of the tests can be related to the respective load–displacement curve and are due to the process of creating new crack surfaces, altering between stages where fibrils are still remaining in the loaded area and sudden crack propagation.

#### 3.5.1. Influence of Surface Energy

PSAs are dependent on the system in which they are constrained, which includes a dependence on the substrate material. Particularly, they also depend on the surface energy of the material. However, acrylic PSAs present poor adhesion on low-surface energy substrates, such as plastics [45]. A typical solution to the wetting problem is to treat the substrate surfaces with corona, plasma, or chemical agents [46]. Thus, with the values obtained from the surface energy measurements, it was expected that the specimens with the plasma treatment presented a higher failure load. However, as shown in Figure 12 with the dashed and solid lines, the difference between them was small, presenting an average value for the failure load of F=51.22N±1.33 for the specimens with no treatment and F=49.99N±1.86 for the specimens with the plasma treatment.

All the fracture surfaces showed an adhesive failure on the second substrate to be bonded, as well as cavitation in the interface between the substrate and the PSA still bonded, after testing. Léger and Creton [47] showed that although the connector molecules in the interface between the PSA and the solid substrate are able to promote adhesion, this dissipation mechanism at a molecular scale is not enough to produce a fracture toughness value that is typical for these materials. There is a need for another energy dissipation mechanism, such as bulk deformation, where a competition between interfacial failure and significant extensional deformations in the bulk adhesive determine the fracture toughness in the end. Considering that this material has characteristics of the non-PSA quadrant of the viscoelastic window, i.e., high elasticity, reduced adhesion, and easy debonding, this PSA may have difficulty flowing sufficiently to create resistant bonds with the second substrate. Thus, after applying pressure, there is less favourable intimate contact between the adhesive and the substrate. Once there is any air entrapment between the layer and the substrate, it is virtually impossible to eliminate them, weakening the interface in question. In their work, Deplace et al. [48] showed that linear rheological measurements, such as determining G′, can be used to have a first idea of adhesive performance. Considering the mentioned results, only the non-treated specimens were considered as a reference when comparing the remaining testing conditions.

Regarding the values of the fracture energy presented in Figure 14 (solid line), the reference specimens obtained an initiation value of approximately 0.9±0.13 N/mm, and then increased until a plateau of about 1.04±0.06 N/mm. The initiation value was related to the failure load in the load–displacement curve with a drop in the load value; however, the specimens continue to increasingly rotate while the fibrils ensure some cohesive strength of the joint, allowing for the fracture energy to increase until the plateau.

#### 3.5.2. Influence of the Roughness

As in the case of surface energy, the substrate roughness affects the performance of the joint, particularly the interfacial cavitation process, which was also concluded by Chiche et al. [49]. When a higher value of the roughness is present, the larger initial defects are being promoted, i.e., the higher peaks promote early interface cavitation. Additionally, the behaviour of the PSA, when comparing different surface roughness values, also depends on the viscosity of the material, which determines its ability to wet all the area, and is enhanced with the peaks and valleys of the high rough surfaces. It is possible to compare the influence of the different values of surface roughness by observing in Figure 12 the solid, dash-dotted, and dotted lines. Specimens DCB_P150, which presented the intermediate roughness, attained an average value of the failure load of F=42.52N±2.66, which consists of a decrease of about 17% when compared with the reference specimen’s value. This result is consistent with the statement that PSAs’ performance is dependent on surface roughness [50]. Specimens DCB_P60, which presented the highest surface roughness, achieved an average value of the failure load of F=43.05N±8.38, which represents an approximate 16% reduction when compared to the reference specimen. However, it should be noted that this roughness resulted in failure loads with higher scatter due to the greater difficulty of the adhesive in wetting a rougher surface and becoming more prone to debond from the substrate. By comparing DCB_P150 and DCB_P60 specimens, a difference of about 1.2% is obtained, which seems to indicate that, for this adhesive, there is not a big difference if the roughness is increased. As for the previous condition, all the fracture surfaces showed an adhesive failure on the second substrate to be bonded, as well as cavitation in the interface between the substrate and the PSA still bonded after testing.

Analysing the fracture energy curves, it is very clear that an increase in the roughness value represents a decrease in the ability of the joint to absorb energy. In Figure 14, with dash-dotted and dotted lines, both DCB_P150 and DCB_60 specimens presented a value for the crack initiation of about 0.6 N/mm, which represents a decrease of 25% when compared to the reference. In average, DCB_P150 attained a value for initiation of J=0.6±0.05 N/mm, while DCB_P60 yielded J=0.6±0.04 N/mm for the same value. Moreover, when comparing between the two different roughness values, although the initiation value is similar, the values for propagation are quite different. DCB_P150 specimens attained an average value of approximately J=0.72±0.03 N/mm, while DCB_P60 specimens gave a value of about J=0.64±0.08 N/mm. This indicates that, although the initiation value is similar for both cases, there is an easier path for the crack to propagate when there is a rougher surface.

#### 3.5.3. Influence of the Number of Stacked Layers

In Section 3.4, it was concluded that when introducing a new interface in a bulk specimen, the tensile strength was highly affected, with a significant decrease in the true tensile strength value. When comparing the load–displacement curve with the reference, in Figure 12, a clear drop in the failure load is also visible. The specimens with two layers achieve an average failure load of F=39.52N±2.10, with a decrease of approximately 23% when compared to the reference. Contrary to the previous conditions, these specimens showed an interfacial failure, between the two layers. Some cavitation in the substrate–adhesive interface was observed, as well as some areas where the adhesive started to debonding from the substrate. Therefore, it is possible to conclude that by introducing a new interface in a DCB specimen, a weaker interface is being added, leading to a decrease in the strength of the joint.

Regarding the fracture energy that can be observed in Figure 14 as a purple solid line, the average value for initiation was about J=0.6±0.07 N/mm, a decrease of 30% when compared to the reference, which is in accordance with introducing a weaker interface. However, this particular condition presented J curves with a slightly different shape. After the initiation, there was a drop in the value, followed by a brief plateau, and then a new increase until the end of the test. This could be explained by the fact that after the initial interfacial failure between the layers, there is a competition between the performance of the two interfaces, resulting in multiple debonded areas, as referred to above. While for the other conditions the values in the load–displacement curves continuously decreased after the failure load, for the two-layer condition, there is a plateau in the curve that occurred while the rotation of the substrates continued to grow, resulting in an increase in the fracture energy values, going from about J=0.41±0.04 N/mm in the plateau region to about J=0.58±0.06 N/mm in the end of the test.

#### 3.5.4. Influence of the Thickness

Finally, the influence of the adhesive thicknesses on the joint performance was evaluated, as can be observed in Figure 12, with the solid cyan line. The failure load, when compared to the reference, presented a huge decrease of about 56%, with an average value of F=22.69N±1.13. With such a thin layer, t=0.13 mm, it is even harder to ensure that there is no air entrapment and complete adhesion to the substrate when producing the joint. As such, during the tests, all the specimens failed in the interface, at very low loads and rotations, indicating a lower adhesion capacity.

When the fracture energy curve is observed, in Figure 14, the reduction compared to the reference specimens is very high. The initiation value decreased to less than J=0.2 N/mm, with an average value of J=0.17±0.02 N/mm and a propagation value of about J=0.27±0.05 N/mm. This seems to indicate that the thinner layer does not perform well in these constraint conditions. This is in line with the results presented by Créton and Ciccotti [51] in their review about fracture and adhesion in soft materials, where it is stated that, for peel tests, the failure mechanisms are dependent on the thickness of the adhesive layer. However, further investigation should be performed to understand the influence of the adhesive layer thickness in the fracture process of PSAs when constrained in a DCB specimen.

## 4. Conclusions

In this work, different properties of an acrylic PSA were determined, as well as the influence of different loading conditions and specimens’ geometry, both for the adhesive and substrate. These properties were determined by resorting to different testing procedures, from mechanical to rheological characterization.

- From the rheological tests it was possible to analyse the viscoelastic behaviour of the PSA and to determine its position in the viscoelastic window. This position allowed for a better understanding of the PSA properties, as well as the adhesive behaviour during testing.
- The tensile tests indicated that the engineering stress and strain should be considered only for small strains, up until 10% for both crosshead speeds (15 mm/min and 225 mm/min). After this strain value, the true values for stress and strain are considerably higher than the engineering ones. Comparing the true tensile strength, it increased almost three times with the higher crosshead speed, resulting in a stronger and more brittle material behaviour.
- The DCB results show that, although the surface energy should improve the performance of the adhesive, the viscoelastic properties of the studied PSA and the test geometry resulted in a similar performance for the treated and non-treated surface. Additionally, the increase in the surface roughness negatively impacted the performance of the joint, with a decrease in the fracture energy values.
- The results from the two stacked layers in the DCB specimens indicate that this new interface becomes the weakest interface, leading to failure between the two layers. However, throughout the test, it seems that the multiple fracture surfaces can contribute to maintain the cohesive strength of the joint.
- The decrease in thickness of the adhesive layer, from 0.26 mm to 0.13 mm, when constrained in DCB specimens, resulted in a bad performance of the joint, with very low fracture energy values.

Although it was possible to find some literature to compare the obtained results, few of them were performed in the same conditions as in the present work. Particularly for DCB testing, the studied conditions were not found in any study, and it could be interesting to continue the investigation in this testing system.

## Figures and Tables

**Table 1:** Roughness average values for the PMMA different surfaces.

Parameter (Values in µm)	Untreated	P150	P60
$R_{a}\;(average)$	1.11 × 10^−3^	1.37 ± 0.07	1.76 ± 0.04
$R_{q}\;(average)$	2.01 × 10^−3^	1.72 ± 0.08	2.23 ± 0.78
$R_{z}(average)$	-	8.44 ± 0.10	11.31 ± 0.10
$R_{max}$	41.50 × 10^−3^	-	-

**Table 2:** Contact angle values for the different liquids in the surface with and without plasma treatment.

Contact Angle (°)			
Surface Treatment	Water	Ethylene Glycol	n-Hexadecane
Without plasma	62.3±0.25	58.1±0.05	12.5±0.5
With plasma	43.6±010	13.2±0.76	0

## Data Availability

The data presented in this study are available on request from the corresponding author. The data are not publicly available due to privacy restrictions.

## References

[1] Sancho-Querol S., Yáñez-Pacios A.J., Martín-Martínez J.M. (2018). New Binary Blends of Ethylene-co-n-butyl Acrylate (EBA) Copolymer and Low Molecular Weight Rosin Ester Resin with Potential as Pressure Sensitive Adhesives. Materials.

[2] Fuensanta M., Martín-Martínez J.M. (2020). Viscoelastic and Adhesion Properties of New Poly(Ether-Urethane) Pressure-Sensitive Adhesives. Front. Mech. Eng..

[3] Fuensanta M., Vallino-Moyano M.A., Martín-Martínez J.M. (2019). Balanced Viscoelastic Properties of Pressure Sensitive Adhesives Made with Thermoplastic Polyurethanes Blends. Polymers.

[4] Sohn S. (2003). Various ways to control the bulk properties of pressure sensitive adhesives. J. Adhes. Sci. Technol..

[5] Chang E.P. (1997). Viscoelastic Properties of Pressure-Sensitive Adhesives. J. Adhes..

[6] Elsmore M.T., Atkinson R.L., Irvine D.J., Howdle S.M., De Focatiis D.S.A. (2022). Sustainable terpene triblock copolymers with tuneable properties for pressure sensitive adhesive applications. Polym. Test..

[7] Gdalin B.E., Bermesheva E.V., Shandryuk G.A., Feldstein M.M. (2011). Effect of Temperature on Probe Tack Adhesion: Extension of the Dahlquist Criterion of Tack. J. Adhes..

[8] Benedek I. (2004). Pressure-Sensitive Adhesives and Applications.

[9] Seok W.C., Leem J.T., Song H.J. (2022). Acrylic pressure-sensitive adhesives based on ethylene glycol acrylate for flexible display application: Highly elastic and recoverable properties. Polym. Test..

[10] Deng X. (2018). Progress on rubber-based pressure-sensitive adhesives. J. Adhes..

[11] Antosik A.K., Mozelewska K., Piątek-Hnat M., Czech Z., Bartkowiak M. (2022). Silicone pressure-sensitive adhesives with increased thermal resistance. J. Therm. Anal. Calorim..

[12] Fuensanta M., Martín-Martínez J.M. (2021). Structural and Viscoelastic Properties of Thermoplastic Polyurethanes Containing Mixed Soft Segments with Potential Application as Pressure Sensitive Adhesives. Polymers.

[13] Lee J.-H., Shim G.-S., Park J.-W., Kim H.-J., Kim Y. (2019). Adhesion performance and recovery of acrylic pressure-sensitive adhesives thermally crosslinked with styrene–isoprene–styrene elastomer blends for flexible display applications. J. Ind. Eng. Chem..

[14] Townsend B.W., Ohanehi D.C., Dillard D.A., Austin S.R., Salmon F., Gagnon D.R. (2011). Characterizing acrylic foam pressure sensitive adhesive tapes for structural glazing applications—Part II: Creep rupture results. Int. J. Adhes. Adhes..

[15] Park K.H., Lee D.Y., Yoon S.H., Kim S.H., Han M.S., Jeon S., Kim Y., Lim Y.K., Hwang D.-H., Jung S.-H. (2022). Adhesion Improvement of Solvent-Free Pressure-Sensitive Adhesives by Semi-IPN Using Polyurethanes and Acrylic Polymers. Polymers.

[16] Seok W.C., Park J.H., Song H.J. (2022). Effect of silane acrylate on the surface properties, adhesive performance, and rheological behavior of acrylic pressure sensitive adhesives for flexible displays. J. Ind. Eng. Chem..

[17] Mapari S., Mestry S., Mhaske S.T. (2021). Developments in pressure-sensitive adhesives: A review. Polym. Bull..

[18] Villey R., Cortet P.-P., Creton C., Ciccotti M. (2017). In-situ measurement of the large strain response of the fibrillar debonding region during the steady peeling of pressure sensitive adhesives. Int. J. Fract..

[19] Pandey V., Fleury A., Villey R., Creton C., Ciccotti M. (2020). Linking peel and tack performances of pressure sensitive adhesives. Soft Matter.

[20] Renvoise J., Burlot D., Marin G., Derail C. (2007). Peeling of PSAs on Viscoelastic Substrates: A Failure Criterion. J. Adhes..

[21] Bartkowiak M., Czech Z., Mozelewska K., Nowak M. (2020). Influence of thermal reactive crosslinking agents on the tack, peel adhesion, and shear strength of acrylic pressure-sensitive adhesives. Polym. Test..

[22] Gurney R.S., Morse A., Siband E., Dupin D., Armes S.P., Keddie J.L. (2015). Mechanical properties of a waterborne pressure-sensitive adhesive with a percolating poly(acrylic acid)-based diblock copolymer network: Effect of pH. J. Colloid Interface Sci..

[23] Tordjeman P., Papon E., Villenave J.-J. (2000). Tack properties of pressure-sensitive adhesives. J. Polym. Sci. Part B Polym. Phys..

[24] Kowalski A., Czech Z. (2015). The effects of substrate surface properties on tack performance of acrylic Pressure-Sensitive Adhesives (PSAs). Int. J. Adhes. Adhes..

[25] Sosson F., Chateauminois A., Creton C. (2005). Investigation of shear failure mechanisms of pressure-sensitive adhesives. J. Polym. Sci. Part B Polym. Phys..

[26] Jimenez N., Ballard N., Asua J.M. (2021). Hydrogen-bond driven formation of microstructured pressure sensitive adhesives (PSAs) with enhanced shear resistance. Polymer.

[27] Huang H., Dasgupta A., Mirbagheri E. Mechanical behavior of pressure-sensitive adhesives (PSAs). Proceedings of the 2017 16th IEEE Intersociety Conference on Thermal and Thermomechanical Phenomena in Electronic Systems (ITherm).

[28] Horgnies M., Darque-Ceretti E., Felder E. (2007). Relationship between the fracture energy and the mechanical behaviour of pressure-sensitive adhesives. Int. J. Adhes. Adhes..

[29] Bartlett M.D., Case S.W., Kinloch A.J., Dillard D.A. (2023). Peel tests for quantifying adhesion and toughness: A review. Prog. Mater. Sci..

[30] Wei Y., Hutchinson J.W., Knauss W.G., Schapery R.A. (1998). Interface strength, work of adhesion and plasticity in the peel test. Recent Advances in Fracture Mechanics: Honoring Mel and Max Williams.

[31] Taub M.B., Dauskardt R.H. (2000). Adhesion of Pressure Sensitive Adhesives with Applications in Transdermal Drug Delivery. MRS Online Proc. Libr. (OPL).

[32] Biel A., Stigh U. (2017). Cohesive zone modelling of nucleation, growth and coalesce of cavities. Int. J. Fract..

[33] Hayashida S., Sugaya T., Kuramoto S., Sato C., Mihara A., Onuma T. (2015). Impact strength of joints bonded with high-strength pressure-sensitive adhesive. Int. J. Adhes. Adhes..

[34] Stigh U., Biel A. (2018). Effects of strain rate on the cohesive properties and fracture process of a pressure sensitive adhesive. Eng. Fract. Mech..

[35] Yang Z., Zhu Z., Yao C., Xia Y., Chen K., Jiang H. (2022). A rate-dependent cohesive zone model for adhesive damage considering fibrillation. Int. J. Mech. Sci..

[36] Benedek I., Feldstein M.M. (2008). Technology of Pressure-Sensitive Adhesives and Products.

[37] (2017). Testing of Rubber—Determination of Tensile Strength at Break, Tensile Stress at Yield, Elongation at Break and Stress Values in a Tensile Test.

[38] Owens D.K., Wendt R.C. (1969). Estimation of the surface free energy of polymers. J. Appl. Polym. Sci..

[39] Chang E.P. (1991). Viscoelastic Windows of Pressure-Sensitive Adhesives. J. Adhes..

[40] Paris A.J., Paris P.C. (1988). Instantaneous evaluation of J and C. Int. J. Fract..

[41] Rice J. (1968). A path independent integral and the approximate analysis of strain concentration by notched and cracks. J. Appl. Mech..

[42] Dantas M.A., Carbas R.J.C., Marques E.A.S., Kushner D., da Silva L.F.M. (2021). Flexible tubular metal-polymer adhesive joints under torsion loading. Int. J. Adhes. Adhes..

[43] Dahlquist C.A. (1966). Adhesion: Fundamentals and Practice.

[44] Huang H., Dasgupta A., Singh N. (2021). Predictive Mechanistic Model of Creep Response of Single-Layered Pressure-Sensitive Adhesive (PSA) Joints. Materials.

[45] Wang Y., Long J., Bai Y., Zhang C., Cheng B., Shao L., Qi S. (2015). Preparation and characterization of fluorinated acrylic pressure sensitive adhesives for low surface energy substrates. J. Fluor. Chem..

[46] Park H.-W., Seo H.-S., Lee J.-H., Shin S. (2020). Adhesion improvement of the acrylic pressure-sensitive adhesive to low-surface-energy substrates using silicone urethane dimethacrylates. Eur. Polym. J..

[47] Léger L., Creton C. (1869). Adhesion mechanisms at soft polymer interfaces. Philos. Trans. R. Soc. A Math. Phys. Eng. Sci..

[48] Deplace F., Carelli C., Mariot S., Retsos H., Chateauminois A., Ouzineb K., Creton C. (2009). Fine Tuning the Adhesive Properties of a Soft Nanostructured Adhesive with Rheological Measurements. J. Adhes..

[49] Chiche A., Pareige P., Creton C. (2000). Role of surface roughness in controlling the adhesion of a soft adhesive on a hard surface. Comptes Rendus De L’académie Des Sci. Ser. IV Phys..

[50] Chiche A., Pareige P., Creton C. (2000). Control of Adhesion through Surface Roughness in a Flat Probe Geometry. Soc. Fr. Du Vide Le Vide Sci. Tech. Et Appl..

[51] Creton C., Ciccotti M. (2016). Fracture and adhesion of soft materials: A review. Rep. Prog. Physics. Phys. Soc..

